# Integrated bioinformatics and machine learning algorithms reveal the critical cellular senescence-associated genes and immune infiltration in heart failure due to ischemic cardiomyopathy

**DOI:** 10.3389/fimmu.2023.1150304

**Published:** 2023-05-10

**Authors:** Ling Guo, Chong-En Xu

**Affiliations:** ^1^ Department of Cardiology, Shandong Provincial Hospital Affiliated to Shandong First Medical University, Jinan, Shandong, China; ^2^ Department of Cardiac Surgery, Shandong Provincial Hospital Affiliated to Shandong First Medical University, Jinan, Shandong, China

**Keywords:** heart failure, cellular senescence, gene, bioinformatics, machine learning

## Abstract

Heart failure (HF) is the final stage of many cardiovascular illnesses and the leading cause of death worldwide. At the same time, ischemic cardiomyopathy has replaced valvular heart disease and hypertension as the primary causes of heart failure. Cellular senescence in heart failure is currently receiving more attention. In this paper, we investigated the correlation between the immunological properties of myocardial tissue and the pathological mechanisms of cellular senescence during ischemic cardiomyopathy leading to heart failure (ICM-HF) using bioinformatics and machine learning methodologies. Our goals were to clarify the pathogenic causes of heart failure and find new treatment options. First, after obtaining GSE5406 from the Gene Expression Omnibus (GEO) database and doing limma analysis, differential genes (DEGs) among the ICM-HF and control groups were identified. We intersected these differential genes with cellular senescence-associated genes (CSAG) *via* the CellAge database to obtain 39 cellular senescence-associated DEGs (CSA-DEGs). Then, a functional enrichment analysis was performed to elucidate the precise biological processes by which the hub genes control cellular senescence and immunological pathways. Then, the respective key genes were identified by Random Forest (RF) method, LASSO (Least Absolute Shrinkage and Selection Operator) algorithms, and Cytoscape’s MCODE plug-in. Three sets of key genes were taken to intersect to obtain three CSA-signature genes (including MYC, MAP2K1, and STAT3), and these three CSA-signature genes were validated in the test gene set (GSE57345), and Nomogram analysis was done. In addition, we assessed the relationship between these three CSA- signature genes and the immunological landscape of heart failure encompassing immunological infiltration expression profiles. This work implies that cellular senescence may have a crucial role in the pathogenesis of ICM-HF, which may be closely tied to its effect on the immune microenvironment. Exploring the molecular underpinnings of cellular senescence during ICM-HF is anticipated to yield significant advances in the disease’s diagnosis and therapy.

## Introduction

Heart failure is a fundamental public health problem affecting millions worldwide, with a 2% - 4% prevalence in developed countries (1). Heart failure is the most common cause of death worldwide, and its prevalence continues to rise as the population ages. In the past few decades, the cause of heart failure has shifted from valvular heart disease and hypertension to coronary artery disease (2). Ischemic heart disease (ICM), caused and progressing by underlying coronary artery disease, is the most common but underestimated cause of heart failure and a significant cause of death in patients with heart failure. Although much research has been done to explore the pathological mechanism of ICM-HF, little is known. The role of cellular senescence in the occurrence and development of heart failure is an essential academic field that has received much attention in recent years. Exploring the pathological mechanism of ICM-HF will bring new insights and ideas for its diagnosis and treatment.

Multiple cellular stressors, including telomere shortening and DNA damage, drive cellular senescence, which is defined by the irreversible cessation of the cell cycle (3). Age-related accumulation of senescent cells in numerous tissues and organs has been associated with age-related diseases, including heart failure (4, 5). This variety of growth factors and pro-inflammatory cytokines, refer to as the senescence-associated secretory phenotype (SASP), can cause damage to neighboring cells and tissue (5, 6).

Recent research demonstrates that senescent cells grow in the heart due to several cardiac stresses, such as myocardial infarction and hypertension (7). These cells are hypothesized to aid in the development of heart failure by increasing fibrosis, inflammation, and oxidative stress (7). However, the molecular and physiological mechanisms underlying the association between cellular senescence and heart failure remain poorly understood.

A complex disease like heart failure is characterized by immunological dysregulation, which causes inflammation and cellular damage. Recent research has demonstrated that immune cells, such as macrophages and T cells, have a significant role in the development and progression of heart failure. In individuals with heart failure, activating pro-inflammatory T lymphocytes and accumulating pro-inflammatory macrophages in the heart have been associated with poor outcomes (8, 9). Targeting the immune system through strategies such as immune cell depletion or modulation of immune cell function may provide a novel therapeutic approach for heart failure.

In general, immunological infiltration and cellular senescence are significant factors that contribute to heart failure, and addressing these mechanisms may offer new therapeutic prospects for treating this disease. Therefore, in this study, we employed an integrated bioinformatics and machine learning strategy to uncover essential genes linked with cellular senescence and alterations in the immunological infiltration in ICM-HF. The findings of this study will provide fresh light on the molecular and cellular research of ICM-HF and emphasize the significance of cellular senescence and immunological dysfunction in the genesis and progression of heart failure conditions. Essential targets for developing new diagnostic and therapeutic techniques for heart failure are provided by identifying critical genes and immunological infiltration related to cellular senescence. To the best of our knowledge, this is the first study to examine the impact of cellular senescence on the myocardial immunological infiltration landscape in ICM-HF using integrated bioinformatics and machine learning algorithms, providing insights into novel therapeutic strategies and a substantial theoretical foundation for future innovative studies.

## Materials and methods

### Data collection

**Figure 1:**
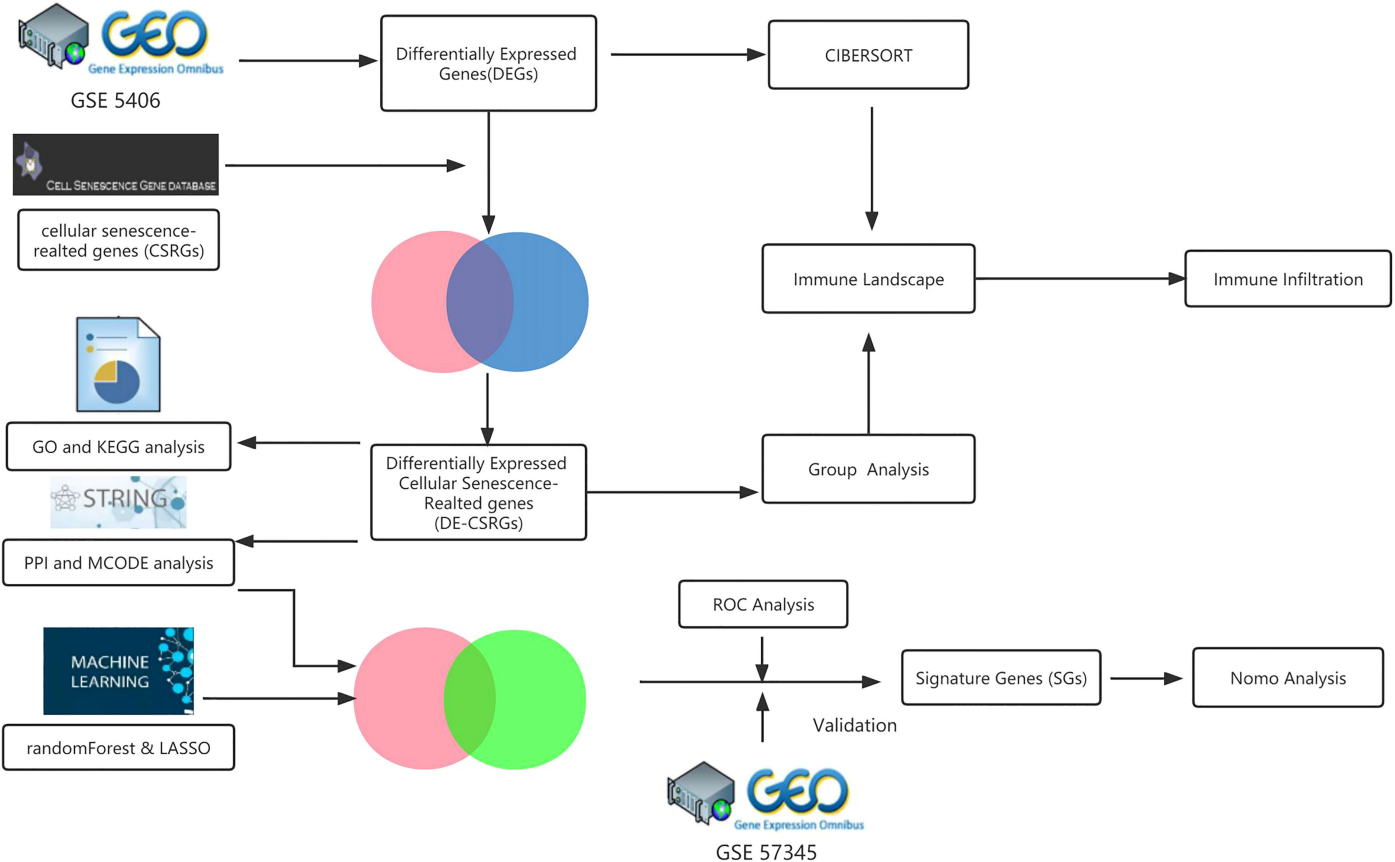
The workflow diagram of the study.

The GEO database was searched for the keyword “heart failure” to find the microarray data adopted in this study. Only datasets that met specific criteria were included in the study, such as human sapiens, array expression profiling, experiments involving heart failure patients due to advanced ischemic cardiomyopathy (ICM) and controls, samples from myocardial tissue, and a sample size of ≥ 20 individuals with ≥ 10 patients in each group. Two datasets, GSE5406 and GSE57345, were ultimately used as the training and test set, respectively. The Cell Senescence Database (http://csgene.bioinfo-minzhao.org/download.cgi) also provided a list of 503 genes associated with cellular senescence. The analytical workflow of this study is illustrated in detail in **Figure 1**.

### Data processing

The investigation employed R statistical software (version 4.1.2) to manage the raw data. The R package Limma (version 3.40.6) was utilized for differential analysis to find DEGs between the ICM-HF and control groups. Cut-off values of p< 0.05 and |log2FC|> 1.2 were used to find DEGs. After eliminating the probes without matching gene symbols, the final expression value was calculated using the average value for several probes mapped to the same gene. The Cell senescence database and the test dataset were used to identify candidate genes.

### Functional enrichment analysis of CSA-DEGs

The online enrichment analysis tool of DAVID 2021 was employed for the functional enrichment analysis of specific DEGs. Gene set enrichment analysis was adopted to determine the enrichment of a specific gene set, and core enrichment genes were identified. We used DAVID resources to perform GO functions (Gene Ontology) and KEGG pathway (Kyoto Encyclopedia of Genes and Genomes) enrichment analysis. We evaluated GO terms in biological processes, molecular functions, and cellular components. GO terms and KEGG maps of biological functions were enriched with a threshold p-value of < 0.05. The “GOplot” R software was used for the visualization (version 1.0.2).

Gene Set Enrichment Analysis (GSEA) was also performed on the DEGs related to cellular senescence using GSEA software (version 4.3.2). The Molecular Signatures Database (MSigDB, version 7.1) reference gene sets “c2.cp.kegg.v7.0. symbols.gmt” and “c5.go.v7.4.symbols” were utilized (10). A criterion of an adjusted q-value of 0.05 was used to assess significance.

### Protein-protein interaction network and potential hub gene analysis

The PPI network was constructed using the String database (available at http://string-db.org) to predict PPI and demonstrate the overarching organizing principles of functional cell systems (11). Using default settings for K nucleus, degree cut-off, node score cut-off, and a maximum depth of 100, The PPI network was analyzed and displayed using the Cytoscape software’s molecular complex detection (MCODE) component. Potential hub genes were found by combining crucial bioinformatics data, including DEGs that satisfied the inclusion requirements (|log2 FC |≥ 1.2 and adjusted p-value < 0.05) and genes with connectivity higher than 5.

### Screening ICM-HF CSA- signature genes by machine learning and MCODE algorithms

The three algorithms were adopted for screening novel and feature biomarkers for ICM-HF, including RF (12), LASSO logistic regression (13), and the MCODE plug-in mentioned above. The LASSO method identifies essential features from large-scale data and selects CSA genes significantly differentially expressed between heart failure and control groups. This is done by using P-value selection and selecting more predictive and less linearly dependent genes. The R package “glmnet” implements LASSO in this process. Random Forest is an ensemble algorithm that aggregates the results of multiple classifiers to get a single decision on behalf of the ensemble (14). Here, we employed the Random Forest technique to predict heart failure by feeding CSA-SGs into the “randomForest” package of the R programming language (14, 15). Then, this study selected overlapping genes from the key genes of the above two machine learning models and the hub genes of the MCODE plug-in as signature genes for further analysis.

### Construction and validation of the nomogram

This study used the previously described methods to identify signature genes associated with cellular senescence to predict heart failure. A nomogram was created using the R package “rms” to denote the ICM-HF incidence based on these signature genes. The “Points” column in the nomogram indicates each gene’s score, while the “Total Points” column displays the total values. Each gene’s “Points” were calculated by tracing a line directly from each gene to the nomogram’s point scale. The risk of ICM-HF was then determined by adding the “Points” and placing them on the “Total Points” scale. A calibration curve was utilized to evaluate the predictive accuracy of the nomogram, and a ROC curve was used to assess the model’s predictive ability.

### Immune infiltration analysis by CIBERSORT algorithm and its correlation with CSA-SGs

To obtain the immune cell matrix, we used the CIBERSORT algorithm to analyze the normalized gene expression matrix and filtered the samples with p<0.05. The “corrplot” package in R was used to calculate the correlation between immune cells and visualize it. Then, the “vioplot” package in R was used to analyze each immune cell’s different levels of immune infiltration between the ICM-HF and control groups. Finally, R’s “ggplot2” package displayed the relationship between three CSA-SGs and different immunocytes.

### Identifying drug candidates

The DSigDB database can be accessed by visiting Enrichr (https://www.amp.pharm.mssm.edu/Enrichr/) website to enter relevant gene targets and download target drug information. Drug candidates are ranked by a combined score from highest to lowest. The combined score represents the degree to which the small molecule drug is closely linked to the gene of interest. Drugs with p-values less than 0.05 and larger combined scores were considered typically significant.

## Results

### Identifying candidate Cell senescence-associated genes in ICM-HF

**Figure 2:**
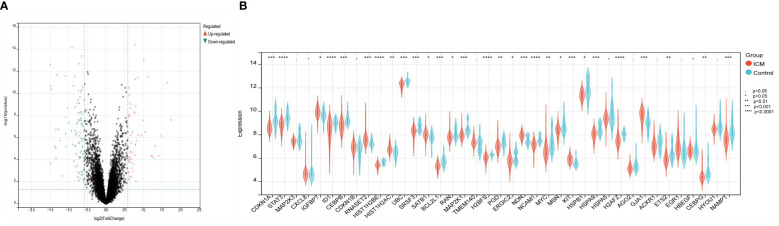
Identification of differentially expressed genes related to cellular senescence. **(A)** Volcano plot visualized the differentially expressed genes (DEGs). In the volcano plot, each dot represents a gene. The red plot points represent up-regulated genes, and the blue plot points represent downregulated genes. **(B)** The difference in the mRNA expression profiling of cellular senescence-associated genes (CSAGs) between the HF and control groups.

The Cell senescence database and the GSE5406 dataset containing left ventricular tissue from 16 healthy controls and 108 patients with left heart failure were used to identify candidate genes. First, 982 differentially expressed genes (442 up-regulated and 540 down-regulated) were identified between left heart failure and healthy samples in the dataset GSE5406 using adjusted P< 0.05 and |log2FC|>1.2 as cut-off values (**Figure 2A**). 982 DEGs were then intersected with 503 CSA genes from the Cell senescence database, identifying 39 CSA-DEGs included in both data sets. Among 39 CSA-DEGs, 10 were up-regulated (IGFBP7, CDKN1B, RNASET2, HIST1H2AC, SATB1, TMEM140, NDN, KIT, GJA1, EGR1) and 29 were down-regulated (CDKN1A, STAT3, MAP2K1, MAP2K3, CXCL8, ID1, CEBPB HIST1H2BE, UBC, SRSF3, BCL2L1, RAN, H2BFS, PGD, ERGIC2, NCAM1, MYC, MSN, HSPB1, HSPA9, HSPA5, H2AFZ, AGO2, ACKR1, ETS2, HBEGF, CEBPG, HYOU1, NAMPT). The expression level of MYC, STAT3, and MAP2K1 was significantly decreased in the ICM group (**Figure 2B**).

### Functional enrichment analysis

**Figure 3:**
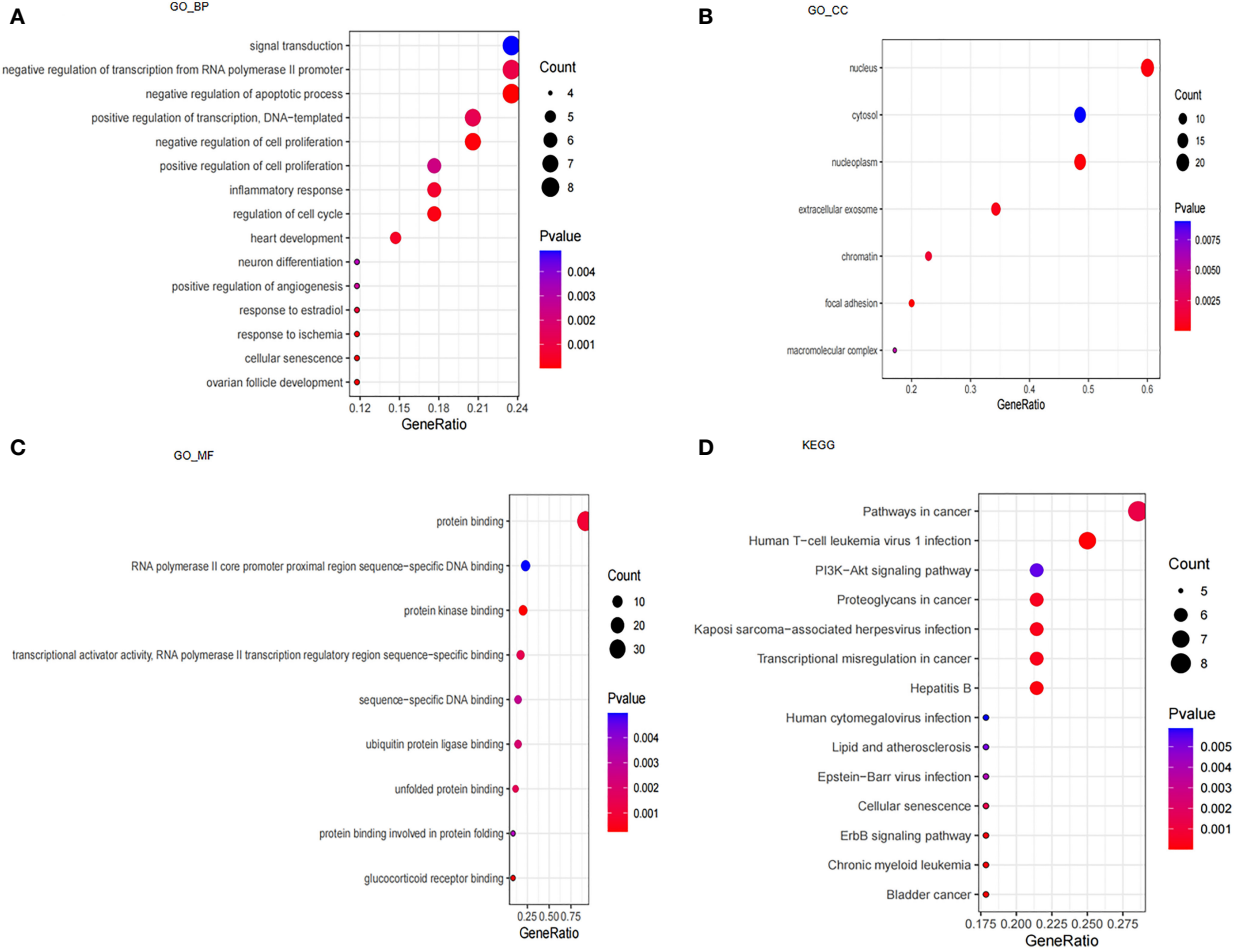
Analysis of GO, KEGG and GSEA. **(A)** GO enrichment analysis based on biological processes (BP). **(B)** GO enrichment analysis based on cellular compartments (CC). **(C)** GO enrichment analysis based on molecular functions (MF). **(D)** KEGG enrichment analysis.

We performed GO and KEGG enrichment analyses to understand the underlying molecular mechanisms of these 39 CSA-DEGs between the ICM-HF and control groups. According to the GO enrichment analysis, these genes were primarily enriched in biological processes, including signal transduction, negative regulation of transcription from RNA polymerase II promoter, negative regulation of apoptotic process, cellular senescence, regulation of cell proliferation, regulation of cell cycle, response to ischemia, and inflammatory response. In cellular components, these genes are mainly enriched in focal adhesion, nucleoplasm, nucleus, extracellular exosome, and chromatin. These genes are also especially abundant in protein kinase binding, glucocorticoid receptor binding, protein binding, transcriptional activator activity, and unfolded protein binding in terms of molecular functions (**Figures 3A–C**). KEGG enrichment analysis showed that 39 CSA-DEGs were significantly activated in the PI3K-Akt signaling pathway, Lipid and atherosclerosis, cellular senescence, and ErbB signaling pathway (**Figure 3D**). These data suggest that cellular aging, immune regulation, and inflammation may play an essential role in the development process of heart failure (**Supplementary Table 1**).

**Figure 4:**
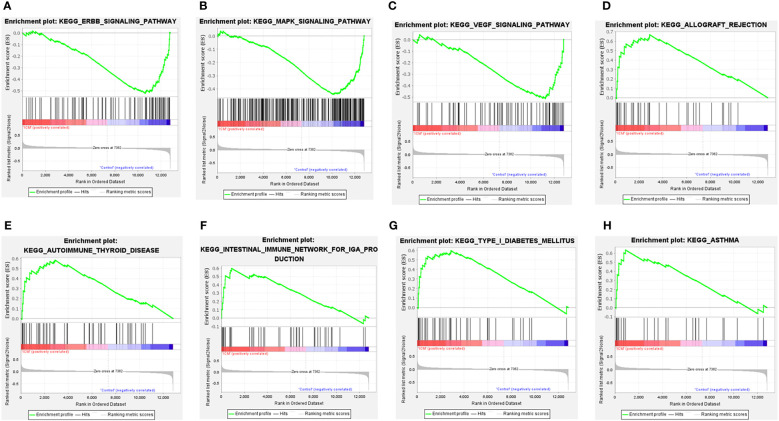
GSEA enrichment analysis of CSA-DEGs between HF and Control tissue samples. **(A, B)** GSEA enrichment analysis results in Control tissue samples. **(C–H)** GSEA enrichment analysis results in ICM- HF tissue samples. ICM, ischemic cardiomyopathy; HF, Heart failure; GSEA, gene set enrichment analysis.

Moreover, GSEA results showed that the ErbB signaling pathway, MAPK signaling pathway, and VEGF signaling pathway were mainly enriched in control samples (**Figures 4A–C**). The allograft rejection, autoimmune thyroid disease, intestinal immune network for IGA production, type I diabetes mellitus, and asthma were mainly enriched in ICM-HF samples (**Figures 4D–H**). These findings suggest that immune response and inflammation may play an essential role in the pathological process of heart failure.

### PPI network construction and hub gene selection

**Figure 5:**
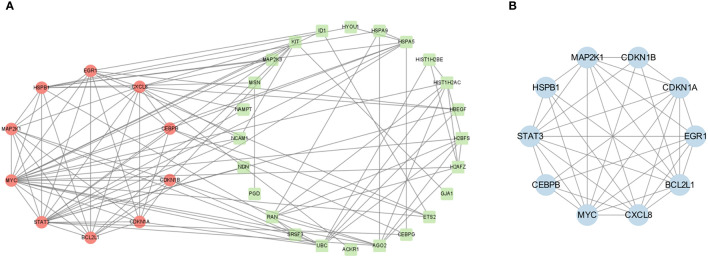
PPI network construction. **(A)** PPI network analysis of CSA- DEGs. The green circles indicate the up-regulated genes, while the red circles represent the down-regulated genes. **(B)** The most significant modules were obtained from the PPI network using MCODE plug-in. Blue color represents genes screened by MCODE plug-in.

PPI analysis of these 39 CSA-DEGs using the String platform was completed, identifying 39 nodes and 107 interactions (**Figure 5A**). The MCODE plug-in was employed to explore the significant modules, and a module with ten nodes with 36 edges was selected from the PPI network (**Figure 5B**). The ten hub genes included STAT3,CDKN1B, HSPB1,BCL2L1, CXCL8, CDKN1A, CEBPB, MAP2K1, MYC,and EGR1. The GO description of MCODE is positive regulation of transcription from RNA polymerase II promoter, negative regulation of apoptotic process, inflammatory response, and cellular senescence.

### Screening ICM-HF CSA- signature genes by machine learning and MCODE algorithms

**Figure 6:**
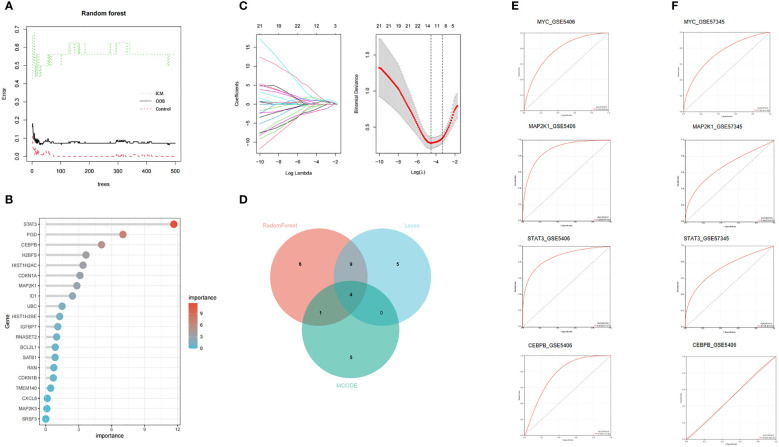
Two machine learning algorithms and MCODE were used for CSA-signature genes. **(A)** The relationship between the number of decision tree and the model error. **(B)** Random Forest (RF) algorithm showed the top 20 candidate genes. **(C)** The LASSO coefficient profiles and 10-fold cross-validation for optimum tuning parameter (l) selection. **(D)** Venn diagram of the intersection of screening results among two machine learning algorithms and MCODE. **(E)** The ROC curves of the HF CSA- signature genes in the training set. **(F)** The ROC curves of the HF CSA- signature genes in the testing set.

Due to the imbalance in the data used in the study, we used SPSSPRO software prior to the machine learning analysis and employed a combined sampling method (SMOTE ENN) to deal with the imbalance in the sample. We applied machine learning methods with the Lasso and Random Forest algorithms to extract 14 and 20 feature genes to assess the diagnostic utility of CSA- DEGs (**Figures 6A–C**). Intersecting the ten hub genes acquired by the MCODE plug-in with the feature genes obtained by the RF and LASSO techniques yielded four CSA- signature genes for heart failure, namely STAT3, MAP2k1, CEBPB, and MYC (**Figure 6D**). To further evaluate the four CSA-signature genes’ diagnostic utility, we created ROC curves and determined the AUC for these signature genes. The findings showed that the AUCs of the four CSA-signature genes varied between 0.78 and 0.89 in the training gene set (**Figure 6E**), demonstrating that these signature genes have outstanding diagnostic power. We performed an external validation of the ROC using the GSE57345 test dataset containing left ventricular tissue from 136 healthy controls and 95 patients with left heart failure. It was found that the AUC of the three remaining CSA signature genes varied between 0.68 and 0.74, except for CEBPB AUC, which was 0.5 (**Figure 6F**), indicating that these three genes also had excellent diagnostic performance in the external test data. The difference in diagnostic performance between two GEO datasets may originate from the cohort difference of different samples. For this reason, we introduced only three CSA signature genes, STAT3, MAP2k1, and MYC, into the follow-up analysis.

### Establishment of the nomogram

**Figure 7:**
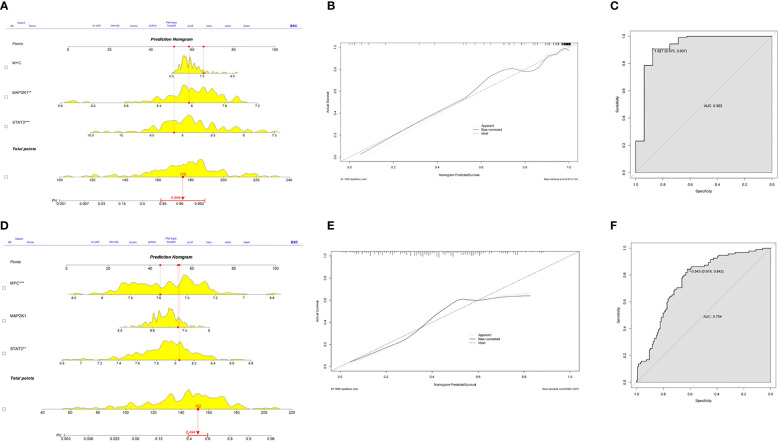
Predictive nomogram to predict the HF risk. **(A)** A nomogram for the training set was developed based on MYC, MAP2K1 and STAT3. **(B)** Calibration test for the training set. **(C)** ROC plot of predictive mode for the training set. **(D)** A nomogram for the test set was validated based on MYC, MAP2K1 and STAT3. **(E)** Calibration test for the test set. **(F)** ROC plot of predictive mode for the test set.

Based on the three CSA- signature genes in the ICM-HF model, we created a predictive nomogram using the GSE5406 dataset (**Figure 7A**), which can be used to determine the likelihood of ICM-HF. Using calibration curves, we evaluated the nomogram’s predictive power, and the outcomes showed that the nomogram was highly accurate in predicting the risk of heart failure (**Figure 7B**). The ensuing ROC evaluation demonstrated that the created diagnostic model has an AUC value of 0.923%, showing this clinical nomogram’s predictive performance (**Figure 7C**). Next, the stability of this nomogram was confirmed using the test data set (GSE57345) (**Figure 7D**). Our nomogram’s precision and stability were further supported by the calibration curve’s proximity to the diagonal (**Figure 7E**) and the AUC value of 0.754 (**Figure 7F**). The results showed that these three CSA-signature genes could distinguish ICM-HF patients from healthy controls.

### Immune infiltration of ICM-HF and its correlation with CSA- signature genes

**Figure 8:**
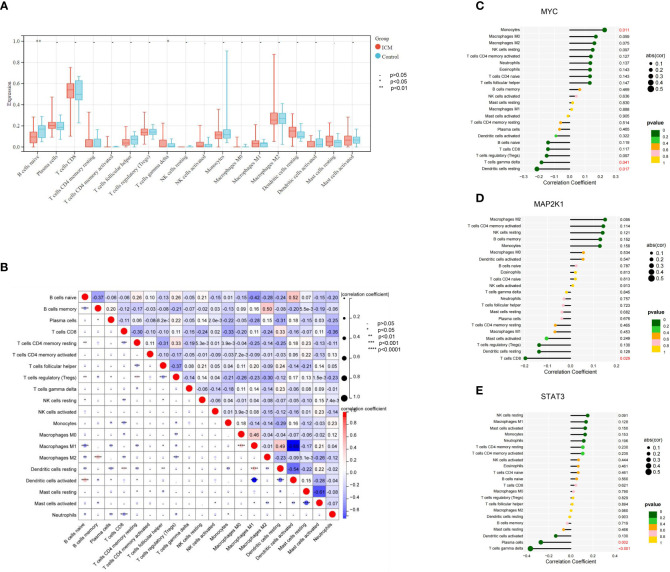
Immune cell infiltration landscape of ICM- HF and its correlation with the CSA- signature genes. **(A)** The distribution of immunocytes between the ICM- HF and control samples. **(B)** The correlation heatmap showed the correlation between different immunocytes in ICM- HF samples. **(C–E)** The lollipop chart showed the correlation between CSA- signature genes and immunocytes in ICM- HF samples.

To understand the immune status in ICM-HF condition, we compared immune infiltration in heart failure patients and controls. Regarding immune cell type, the ICM-HF groups had significantly fewer naive B cells (P < 0.05) than the healthy control. Meanwhile, γ/δ T cells in the ICM-HF patients increased considerably (P < 0.05) (**Figure 8A**). According to Spearman correlation analysis, Macrophage M1 was strongly negatively connected with Dendritic cells activated cells and much positively correlated with B cells naive in ICM-HF patients (P < 0.05) (**Figure 8B**). We subsequently looked into how the ICM-HF CSA- signature genes interacted with the infiltrating immune cells in our samples. We found that MYC and Macrophage M0, as well as γ/δ T cells, MAP2K1 and T cells CD8, and STAT3 and γ/δ T cells, were the pairs of ICM-HF CSA-signature gene-immune (P < 0.05) (**Figures 8C–E**).

### Identification of candidate drugs

The study further involved 3 CSA-signature genes that were used to screen potential drugs for the treatment of HF. According to the combined score, the top 10 candidate drugs were generated (**Table 1**). Deguelin, Sorafenib, celecoxib, staurosporine, Capsaicin, rapamycin, PD 98059, N-Acetyl-L-cysteine, TAUROCHOLIC ACID, and AZOXYMETHANE BOSS were identified by the three CSA-signature genes. Of the top ten drug candidates, only three have been approved by the US Food and Drug Administration, including Sorafenib, celecoxib, and Capsaicin.

**Table 1:** Top 10 drugs analyzed by Enrichr tool.

Term	Adjusted P-value	Combined Score	Genes
Deguelin CTD 00003487	4.43E-05	964206.8544	MAP2K1;MYC;STAT3
Sorafenib CTD 00004146	4.43E-05	928027.5193	MAP2K1;MYC;STAT3
celecoxib CTD 00003448	4.43E-05	923156.8193	MAP2K1;MYC;STAT3
staurosporine CTD 00007273	1.05E-04	851941.3765	MAP2K1;MYC;STAT3
Capsaicin CTD 00005570	1.51E-04	815991.6098	MAP2K1;MYC;STAT3
rapamycin CTD 00007350	2.17E-04	781887.9915	MAP2K1;MYC;STAT3
PD 98059 CTD 00003206	2.22E-04	770779.5725	MAP2K1;MYC;STAT3
N-Acetyl-L-cysteine CTD 00005305	3.38E-04	735956.2776	MAP2K1;MYC;STAT3
TAUROCHOLIC ACID CTD 00006831	3.49E-04	21384.7246	MYC;STAT3
AZOXYMETHANE BOSS	6.60E-04	13587.33897	MYC;STAT3

## Discussion

Heart failure is the ultimate outcome of all cardiovascular diseases and a major cause of mortality in the global population, with a significant impact on the quality of life and disability of patients. Revealing the mechanism of heart failure can effectively improve the effectiveness of treatment for heart failure. Clinical evidence suggests a close relationship between cell aging and worse treatment outcomes for heart failure in most cases. There is also evidence that heart failure is closely related to immune inflammation. This provides new ideas and entry points for the treatment of heart failure. Therefore, controlling cellular aging and anti-inflammatory, immune regulatory strategies may become the most attractive methods for treating heart failure.

In summary, exploring the mechanisms of cellular senescence and immune infiltration related to the occurrence and development of heart failure is of great significance for an in-depth understanding of the pathological mechanism of heart failure, early diagnosis, and identifying new therapeutic targets from the perspective of cellular senescence and immune regulation. To our knowledge, this was the first study to comprehensively explore the involvement of CSAG in the pathological mechanism of heart failure by integrated bioinformatics analyses. A diagnostic model was further developed based on three CSA- signature genes for heart failure.

We initially examined the expression and functions of CSAG in heart failure. 39 CSAG were significantly expressed differently in heart failure compared to controls. These genes were primarily associated with cell senescence, negative apoptosis, regulation of cell proliferation, and inflammation response. Consistent with this finding, features of cell senescence are frequently described by irreversible proliferation arrest, resistance to apoptosis, and a pro-inflammatory secretory phenotype (16). Moreover, these genes were also involved in pathways, such as ErbB signaling and PI3K-Akt signaling pathway. PI3K/AKT pathway plays a critical role in aging (17), and its dysregulation is closely implicated in myocardial fibrosis, a leading driving factor for the progress of heart failure (18, 19).

Interestingly, our GSEA analysis indicated that ErbB signaling was inactivated in heart failure compared to healthy control, suggesting that ErbB signaling might be an important pathway in heart failure. ErbB signaling is essential in cardiac development and cardiovascular homeostasis, which serve as receptors mediating the biological effects of neuregulin-1 (NRG-1), a protective factor in heart failure (20, 21). Pharmacological activation of ErbB signaling or proteins in ErbB signaling has been proposed as potential drug targets for treating heart failure (22, 23). In summary, we speculated that these dysregulated CSAG might be involved in the pathogenesis of heart failure by mediating these signaling pathways.

Next, we used LASSO and RF algorithms and MCODE plug-in to identify three robust potential biomarkers as CSA- signature genes (including MYC, MAP2k1, and STAT3) from 39 CSA-DEGs. It has been previously demonstrated that MYC, MAP2K1, and STAT3 all have a critical role in the development of heart failure. MAPK signaling cascades are believed to be essential effectors of cardiomyocyte mortality in response to various stressors, including ischemia-reperfusion injury. Extracellular signal-regulated kinase 1/2 (ERK1/2) activity is linked explicitly to cardioprotection, possibly through the inhibition of apoptosis-regulate pathways (24). MAP2K125 encodes mitogen-activated protein kinase 1 (MEK1) (25). *In vivo*, genetic suppression of cardiac ERK1/2 enhances apoptosis triggered by stress and heart failure (26). Additionally, MEK has been linked to cardiac ischemia illness, where it promotes ERK1/2 signaling to shield the heart from ischemic damage. Senescent cardiomyocytes and aged hearts benefited from the activation of ERK1/2 signaling because it balanced the fusion and fission of mitochondria in response to ischemia (27).

STAT3 is a crucial signaling molecule vital in HF development and its relationship with cellular senescence. In mice treated with ISO, S31-201, a selective STAT3 inhibitor, nearly eliminated the increase in ventricular dysfunction caused by IL-9. According to these findings, STAT3 activation is needed for IL-9 to cause inflammation in the heart, which may be a cause of HF caused by ISO (28). The inactivation of STAT3 by gp130 loss in pressure-overloaded hypertrophy model mice increased cardiac failure (29). Furthermore, in human cardiac progenitor cells treated with rapamycin, the STAT3-Pim1 axis influences cellular senescence and differentiating capacity (30).

The transcription factor C/EBPβ has been reported to play an important role in physiological hypertrophy and heart failure (31). c/EBPβ inhibits the growth and proliferation of cardiomyocytes. Reducing C/EBPβ expression exacerbates heart failure during pressure overload (31). C/EBPβ is involved in the tumor development process by regulating metabolism (32) and the TGFβ signaling pathway (33). C/EBP is a CCAAT/enhancer binding protein transcription factor that regulates cell growth and differentiation. Previous results suggest a protective effect of C/EBPβ on pathological cardiac remodeling (31). C/EBPβ is also a major regulator of metabolic pathways and insulin resistance (32). All these reports imply a potential role of C/EBPβ in the development of heart failure. Although CEBPB failed to enter the range of important CSA signature genes in our study, its role in the development of heart failure cannot be ignored. MYC is an oncogene that can regulate various key cellular functions, such as regulating metabolism (34) and RNA splicing (35). Transcriptional paused release in heart failure is mediated by BET bromodomain (36), and BET bromodomain inhibitors can inhibit the development of heart failure by regulating innate inflammatory networks (37). All these results suggest a potentially important role of MYC in heart failure.

Previous reports have found that C/EBPβ, MYC, and their target genes are downregulated in failing heart tissue (38). This is consistent with our findings. It has been shown that the insulin signaling pathway is inactivated during the development of heart failure, and MAP2K1 is a key downstream gene of the insulin signaling pathway (39). The heart is dependent on the insulin signaling pathway for metabolism, growth and survival (40). Deficiency of the insulin signaling pathway leads to cardiac energy deficiency and accelerates the progression of heart failure (41). In addition, transcription factors are usually major regulators of disease, regulating multiple target genes. Through a literature search, we identified previously reported MYC target genes, including STAT3 (30). MYC’s role in regulating heart failure development is quite complex. Previous reports have suggested that inhibition of MYC is a potential therapeutic approach for treating hypertrophic cardiomyopathy (42). However, we observed that MYC expression and the target gene of MYC were downregulated in failing heart tissue. This inconsistency further emphasizes the complexity of the MYC-regulated transcriptional network and heart failure progression.

ROC curves indicated that these three CSA- signature genes exhibit good discrimination ability for heart failure in both training and validation datasets. Furthermore, we established an ICM-HF risk prediction nomogram based on three CSA- signature genes, which would help differentiate individuals at high risk of developing heart failure. Such a nomogram could guide the implementation of preventative measures and identify individuals who would benefit from early intervention. Building a nomogram is a complicated process requiring a significant sample size and sufficient data to validate the nomogram. The nomogram should be validated in external independent data sets and evaluated for its discriminatory ability and clinical utility. The AUC value of our nomogram in the training set (GSE5406) is 0.923, whereas, in the testing set (GSE57345), it is 0.754, which further confirms the diagnostic potential of these CSA- signature genes for heart failure patients. The difference in these two AUC values may be derived from different cohort data.

This study identified ten drug molecules by 3 CSA-signature genes, but the FDA approved only three. These three drugs’ main action pathways are cell cycle regulation, cellular senescence, inflammatory response, negative regulation of cell proliferation, and positive regulation of gene expression. However, these three drugs have not been used previously for treating ischemic heart failure, and the therapeutic effects of these three drug candidates on ischemic heart failure need further study.

Studies from our and other organizations indicate that immune infiltration is crucial in HF development. There is accumulating evidence that immune cell infiltration of the myocardium impairs cardiac function (43–45). Single-cell sequencing analysis has shown that the immune cell profile significantly differs in healthy and heart failure hearts (46–48). A more profound comprehension of the function of each type of infiltrating immune cells could provide clues for the creation of novel therapeutic techniques for heart failure.

Due to the complicated composition of immune cells and the interplay between immune functions, traditional research methodologies have been unable to replicate the immunological landscape in myocardial tissue in heart failure in a systematic manner. This time, we analyzed immune infiltration in HF and control patients using CIBERSORT. Compared to other immune cells, CD8+ T cells, M2 macrophage, and plasma cells showed high infiltration abundance in heart failure and healthy myocardial tissue. Infiltration abundance of naive B cells and gamma delta (γ/δ) T cells significantly differed in heart failure from healthy controls.

γ/δ T cells are non-traditional lymphocytes with numerous innate immune cell traits and are the body’s first line of defense against sterile and non-sterile inflammation. γ/δ T cells are not only involved in maintaining the epithelial barrier but also produce a variety of cytokines. These cytokines coordinate the immune response process and exert high cytotoxic activity against infected and transformed cells. One study has demonstrated that lower absolute (worse) left ventricular global circumferential strain was associated with higher proportions of γ/δ T cells (49). This is consistent with our findings. γ/δ T cells are implicated in anti-infectious actions and the genesis and progression of autoimmune disorders (50). Two publications have successively reported the occurrence of dilated cardiomyopathy mediated by γ/δ T cells infiltration (51) and the event of aortitis and heart failure induced by γ/δ T cells activation (52). In myocardial tissue damaged by γ/δ T cells activation in heart failure, high expression of some molecules associated with γ/δ T cells, including perforin, human leukocyte antigens class I and II, and intercellular adhesion molecule-1, could be found in the myocardium (52). And two other studies showed that γ/δ T cells could promote myocardial inflammation (53, 54). Earlier published mechanistic studies suggest that γ/δ T cells enhance the infiltration of neutrophils and macrophages into the myocardial tissue through pathways related to IL-1β, IL-17, and upregulation of pathogenic Th1 responses (55, 56).

B lymphocytes exhibit close associations with the heart, which are demonstrated to be crucial modulators for myocardial adaptation to injury in the context of pathology (57, 58). The myocardium contains more B cells than the epicardium, and the interstitium is more productive than the intravascular space. Myocardial B cells are primarily naive B cells (59, 60). Adhesion of a sub-population of naïve circulating B cells on myocardial microvascular endothelium has been reported recently (58). Infusion of bone marrow B cells could improve myocardial injury post myocardial infarction, during which marrow naive B cells were the significant subset in response to an acute myocardial injury (61). In this study, we observed significantly different infiltration abundance of naive B cells in myocardial tissue of heart failure and healthy controls. Such differences were also observed in the study of Kong et al. (62).

We further analyzed the correlations between immune cells and three CSA- signature genes and revealed a significant negative correlation between γ/δ T cells and STAT3. STAT3 is considered an essential critical cell-intrinsic regulator for the number and function of unconventional T cells. For example, STAT3 is required for IL-17 secretion by γ/δ T cells, and little IL-17 production from STAT3-mutated γ/δ T cells (63). STAT3 is required for inflammatory γ/δT17 cell responses and cytokine production from γ/δT17 cells during inflammation but is not necessary at a steady state (64). These highlight the associations between γ/δ T cells and STAT3. Although a significant negative correlation between γ/δ T cells and STAT3 was observed in this study, a causal relationship could not explain this. But, we can reasonably speculate that downregulation of STAT3 may affect the function of γ/δ T cells in response to inflammation.

Moreover, expression changes of STAT3 could indicate the alternation of γ/δT cell infiltration in myocardial tissue. Such alternation was implicated in myocardial inflammation and injury, further suggesting the potentiality of STAT3 as a diagnostic biomarker. Similarly, expression of these three CSA- signature genes also showed correlations with other important immune cells in heart failure, such as Tregs and CD8^+^ T cells. For example, Th17/Treg imbalance, mainly by reduced Tregs, while elevated Th17 cells, could aggravate myocardial fibrosis and heart failure (65). Depletion of CD8+T cells could minimize ischemic cardiomyocyte death and myocardial damage while inducing cardioprotective hypertrophy. CD8+T cells can regulate the conversion of cardiac-resident macrophages (66, 67).

Inflammation and heart failure are closely related and mutually reinforcing. Inflammation in the body can take many different forms, including sterile inflammation, para-inflammation, and chronic inflammation, all of which have been proven to be strongly linked to the onset of heart failure. Among them, the ischemia-induced sterile inflammatory response is similar to during infection. It can be characterized by the recruitment of neutrophils and macrophages (mφs), the production of inflammatory cytokines and chemokines, and the induction of T cell-mediated adaptive immune responses (68). Parainflammation is mainly dependent on tissue-resident macrophages. If tissue dysfunction persists for some time, para-inflammation may become chronic (69). There is growing evidence that chronic inflammation induces myocardial myofibroblasts to express chemokines that attract immune cells to the heart; cause adhesion molecules on the endothelium; stimulate monocytes to express gelatinase (70, 71); and release NLRP3 inflammatory vesicle activity and IL-1ß (72). In this way, a vicious cycle of chronic inflammation induction is generated in cardiac tissue. In our study, MYC expression was positively correlated with macrophages in ICM-HF myocardial tissue. Therefore, we can speculate that up-regulation of MYC may increase macrophage aggregation in heart failure myocardium.

### Study limitation

However, our research results also have limitations that should be considered when evaluating conclusions. First, Our research results are derived from currently available gene expression datasets, including microarray and RNA-seq data. Despite the possibility that some genes contribute to the formation and progression of ICM-HF, their lack of technical sophistication has led us to neglect them in our research. Second, the data samples used for this study are from human cardiac tissue, not from peripheral blood. Therefore, whether the potential ICM-HF-related diagnostic biomarkers mined using machine learning algorithms are suitable for blood samples requires further observation and verification. Thirdly, although external data sets have validated the three potential ICM-HF biomarkers, validating these results using cardiac tissue from ICM-HF patients is crucial and urgent. Finally, due to incomplete information in the dataset, clinical data in the nomogram was excluded. Adding clinical information closely related to heart failure to the nomogram will improve the accurate screening and diagnosis of ICM-HF.

### Conclusion

In conclusion, the current study sheds new light on the pathological mechanisms of heart failure by investigating the roles of cellular aging and the immunological environment in HF progression. Our findings suggest that cellular senescence and immunological dysregulation are linked to the clinical reasons of heart failure, potentially opening up new therapeutic avenues for HF treatment. The study also indicates that the three CSA-signature genes (MYC, MAP2K1, and STAT3) may not only act as potential biomarkers for heart failure but also be potential targets for future research in the development of new treatment strategies for heart failure.

## Data availability statement

The datasets presented in this study can be found in online repositories. The names of the repository/repositories and accession number(s) can be found below: https://www.ncbi.nlm.nih.gov/geo/query/acc.cgi?acc=GSE5406 and GSE57345.

## Author contributions

Bioinformatic and statistical studies were undertaken by C-EX, and LG. C-EX authored the article. C-EX and LG designed the experiments and analyzed the results. The paper has been read and approved by all authors.
