# Supplementary material for: Integrated bioinformatics and machine learning algorithms reveal the critical cellular senescence-associated genes and immune infiltration in heart failure due to ischemic cardiomyopathy
Source: Front Immunol. 2023 May 10;14:1150304. doi: 10.3389/fimmu.2023.1150304 (PMC10206252; doi:10.3389/fimmu.2023.1150304)
Supplement: Supplementary file 1 [file Table_1.docx]

Supplemetary Table 1 The Function Enrichment Analysis by David tool

| Category and Term | Description | Count | PValue | Genes |
| --- | --- | --- | --- | --- |
| GOTERM_BP_DIRECT | | | | |
| GO:0010628 | positive regulation of gene expression | 9 | 2.14E-06 | EGR1, MAP2K1, GJA1, CXCL8, MYC, ID1, STAT3, AGO2, MSN |
| GO:0045944 | positive regulation of transcription from RNA polymerase II promoter | 9 | 7.82E-04 | EGR1, CEBPB, MYC, STAT3, NAMPT, AGO2, CEBPG, NDN, ETS2 |
| GO:0043066 | negative regulation of apoptotic process | 8 | 2.57E-05 | HSPA9, CDKN1A, CDKN1B, HSPA5, MYC, ID1, HSPB1, BCL2L1 |
| GO:0000122 | negative regulation of transcription from RNA polymerase II promoter | 8 | 0.001081764 | EGR1, CEBPB, SATB1, MYC, ID1, STAT3, NDN, ETS2 |
| GO:0007165 | signal transduction | 8 | 0.004857256 | MAP2K3, MAP2K1, GJA1, CXCL8, STAT3, NAMPT, KIT, HBEGF |
| GO:0008285 | negative regulation of cell proliferation | 7 | 1.18E-04 | CDKN1A, MAP2K1, CDKN1B, CXCL8, STAT3, NDN, IGFBP7 |
| GO:0045893 | positive regulation of transcription, DNA-templated | 7 | 0.001259665 | MAP2K3, EGR1, MAP2K1, CEBPB, MYC, STAT3, ETS2 |
| GO:0051726 | regulation of cell cycle | 6 | 2.07E-04 | CDKN1A, MAP2K1, CDKN1B, CXCL8, MYC, STAT3 |
| GO:0006954 | inflammatory response | 6 | 6.29E-04 | MAP2K3, CEBPB, CXCL8, STAT3, KIT, ACKR1 |
| GO:0008284 | positive regulation of cell proliferation | 6 | 0.002300253 | CDKN1B, MYC, NAMPT, KIT, BCL2L1, HBEGF |
| GO:0007507 | heart development | 5 | 5.07E-04 | MAP2K3, CDKN1A, MAP2K1, GJA1, CDKN1B |
| GO:0071480 | cellular response to gamma radiation | 4 | 1.97E-05 | EGR1, CDKN1A, HSPA5, BCL2L1 |
| GO:0090398 | cellular senescence | 4 | 1.06E-04 | MAP2K3, CDKN1A, MAP2K1, CDKN1B |
| GO:0002931 | response to ischemia | 4 | 1.76E-04 | MAP2K3, EGR1, GJA1, HYOU1 |
| GO:0032355 | response to estradiol | 4 | 9.10E-04 | GJA1, CDKN1B, MYC, STAT3 |
|  |  |  |  |  |
| GOTERM_CC_DIRECT | | | | |
| GO:0005634 | nucleus | 21 | 3.00E-04 | EGR1, CDKN1A, MAP2K1, CEBPB, CDKN1B, HSPA5, SATB1, STAT3, |
|  |  |  |  | CEBPG, HSPB1, MSN, PGD, ETS2, GJA1, MYC, ID1, UBC, AGO2, |
|  |  |  |  | NDN, ERGIC2, RAN |
| GO:0005654 | nucleoplasm | 17 | 2.17E-04 | MAP2K3, EGR1, CDKN1A, CEBPB, CDKN1B, SATB1, STAT3, CEBPG, |
|  |  |  |  | ETS2, GJA1, MYC, ID1, UBC, AGO2, NDN, SRSF3, RAN |
| GO:0005829 | cytosol | 17 | 0.008991195 | MAP2K3, CDKN1A, MAP2K1, CDKN1B, HSPA5, STAT3, HSPB1, MSN, |
|  |  |  |  | PGD, ETS2, UBC, NAMPT, AGO2, NDN, NCAM1, RAN, BCL2L1 |
| GO:0070062 | extracellular exosome | 12 | 6.05E-04 | HSPA9, HSPA5, RNASET2, UBC, NAMPT, AGO2, HSPB1, MSN, |
|  |  |  |  | IGFBP7, HYOU1, PGD, RAN |
| GO:0000785 | chromatin | 8 | 0.001483697 | EGR1, CEBPB, SATB1, MYC, STAT3, CEBPG, ETS2, RAN |
| GO:0005925 | focal adhesion | 7 | 6.17E-05 | HSPA9, MAP2K1, GJA1, HSPA5, HSPB1, MSN, HYOU1 |
| GO:0032991 | macromolecular complex | 6 | 0.005612 | CDKN1A, CDKN1B, HSPA5, MYC, NDN, RAN |
|  |  |  |  |  |
| GOTERM_MF_DIRECT | | | | |
| GO:0005515 | protein binding | 32 | 9.52E-04 | CDKN1A, CEBPB, CDKN1B, CXCL8, SATB1, CEBPG, HSPB1, TMEM140, |
|  |  |  |  | ETS2, GJA1, MYC, UBC, NAMPT, NDN, NCAM1, IGFBP7, MAP2K3, |
|  |  |  |  | HSPA9, EGR1, MAP2K1, HSPA5, STAT3, MSN, ID1, AGO2, KIT, |
|  |  |  |  | SRSF3, HYOU1, ERGIC2, RAN, BCL2L1, HBEGF |
| GO:0000978 | RNA polymerase II core promoter proximal region sequence-specific DNA binding | 8 | 0.004984304 | EGR1, CEBPB, SATB1, MYC, STAT3, CEBPG, NDN, ETS2 |
| GO:0019901 | protein kinase binding | 7 | 2.67E-04 | MAP2K3, CDKN1A, CDKN1B, STAT3, HSPB1, MSN, BCL2L1 |
| GO:0001228 | transcriptional activator activity, RNA polymerase II transcription regulatory region sequence-specific binding | 6 | 0.001535612 | EGR1, CEBPB, MYC, STAT3, CEBPG, NDN |
| GO:0031625 | ubiquitin protein ligase binding | 5 | 0.002152756 | HSPA9, CDKN1A, CDKN1B, HSPA5, UBC |
| GO:0043565 | sequence-specific DNA binding | 5 | 0.002795462 | EGR1, SATB1, MYC, CEBPG, ETS2 |
| GO:0051082 | unfolded protein binding | 4 | 0.001662262 | HSPA9, HSPA5, HSPB1, HYOU1 |
| GO:0044183 | protein binding involved in protein folding | 3 | 0.003500586 | HSPA9, HSPA5, HSPB1 |
| GO:0035259 | glucocorticoid receptor binding | 3 | 3.25E-04 | CEBPB, STAT3, ETS2 |
|  |  |  |  |  |
| KEGG_PATHWAY | | | | |
| hsa05166 | Human T-cell leukemia virus 1 infection | 7 | 6.74E-05 | EGR1, CDKN1A, MAP2K1, MYC, ETS2, RAN, BCL2L1 |
| hsa05167 | Kaposi sarcoma-associated herpesvirus infection | 6 | 3.71E-04 | CDKN1A, MAP2K1, CXCL8, MYC, UBC, STAT3 |
| hsa04151 | PI3K-Akt signaling pathway | 6 | 0.005352276 | CDKN1A, MAP2K1, CDKN1B, MYC, KIT, BCL2L1 |
| hsa05163 | Human cytomegalovirus infection | 5 | 0.005884802 | CDKN1A, MAP2K1, CXCL8, MYC, STAT3 |
| hsa05417 | Lipid and atherosclerosis | 5 | 0.005011468 | MAP2K3, CXCL8, HSPA5, STAT3, BCL2L1 |
| hsa04012 | ErbB signaling pathway | 5 | 1.57E-04 | CDKN1A, MAP2K1, CDKN1B, MYC, HBEGF |
| hsa05169 | Epstein-Barr virus infection | 5 | 0.004013369 | MAP2K3, CDKN1A, CDKN1B, MYC, STAT3 |
| hsa04218 | Cellular senescence | 5 | 0.00156902 | MAP2K3, CDKN1A, MAP2K1, CXCL8, MYC |
| hsa04912 | GnRH signaling pathway | 4 | 0.003386025 | MAP2K3, EGR1, MAP2K1, HBEGF |
| hsa01522 | Endocrine resistance | 4 | 0.00392555 | CDKN1A, MAP2K1, CDKN1B, HBEGF |
| hsa04933 | AGE-RAGE signaling pathway in diabetic complications | 4 | 0.004155224 | EGR1, CDKN1B, CXCL8, STAT3 |
| hsa04928 | Parathyroid hormone synthesis, secretion and action | 4 | 0.004892923 | EGR1, CDKN1A, MAP2K1, HBEGF |
| hsa04066 | HIF-1 signaling pathway | 4 | 0.005289664 | CDKN1A, MAP2K1, CDKN1B, STAT3 |
| hsa04068 | FoxO signaling pathway | 4 | 0.008793171 | CDKN1A, MAP2K1, CDKN1B, STAT3 |
